# Pragmatism or idealism: a systematic review and visual analysis of Winnicott’s psychoanalytical treatment views

**DOI:** 10.3389/fpsyt.2023.1237005

**Published:** 2023-07-28

**Authors:** Zhengyan Xie, Yuting Yan, Kejuan Peng

**Affiliations:** ^1^School of International Studies, Hunan Institute of Technology, Hengyang, Hunan, China; ^2^School of Humanities, Universiti Sains Malaysia, Pulau Pinang, Malaysia; ^3^Wanshou Road Community Health Service Center, Beijing, China

**Keywords:** Winnicott, psychoanalysis, mentality, emotion, treatment

## Abstract

Winnicott is an outstanding representative of the School of Object Relations, and his unique psychoanalytic treatment views have been greatly influential to the psychoanalytical community. Winnicott emphasizes the impact of facilitating environment and the key role of the maternal-child relationship in the early psychological growth of individuals. He puts forward the ideas of the development mechanism of the true self and the false self, which builds a bridge between the characteristics of adult psychopathology and the characteristics of early maternal-child relationships, providing a new perspective for research on individual self-development and psychoanalysis. Winnicott creatively introduces the concepts of the transitional object and the transitional phenomena into the theories of Object Relations. He relates the transitional experience to the field of mental health, and extends it from the relationship between the mother and the child to adult life, which not only has had a revolutionary impact on modern psychoanalysis but also literature, aesthetics, and other fields. Winnicott highlights the importance of the patient’s emotional development in the treatment. He advocates holding the patients’ sentiments and meeting their emotional needs. He also approves of the emotional reparenting of the patients, to make them gain the ability to establish a relationship with the real world. His treatment views formed through a large number of clinical practices are very practical and full of humanistic care. This review summarizes Winnicott’s psychoanalytical treatment views as well as his marvelous original concepts, and analyzes the hot topics of academic research on his theories based on a visualization analysis by using the software CiteSpace, which includes data in the Web of Science Core Collection published from 1978 to 2023 with 365 papers involved. The study provides a macroscopic and panoramic review of Winnicott’s theories, and it clearly shows Winnicott’s significant influence on the field of psychoanalysis and related fields.

## 1. Introduction

D.W. Winnicott (1–3), a well-known British psychoanalyst, pediatrician, and President of the British Psychoanalytical Society twice, is an outstanding representative of the School of Object Relations. He has made enormous contributions and great influence in psychoanalysis, child parenting, mental health, and other related fields. His series of theories on individual growth, such as good-enough mother, facilitating environment, transitional object, true self, and false self, etc., are innovative concepts deeply rooted in psychoanalytic theory. With 40 years of clinical work at the Paddington Green Children’s Hospital in London, he has accumulated rich practical experience and developed a unique thinking of psychoanalytic treatment, which has a profound impact on the field of psychoanalysis.

## 2. An overview of Winnicott’s psychoanalysis

### 2.1. The concept of psychoanalysis

Winnicott points out that psychoanalysis is a method of using psychological methods to treat the mentally ill. Psychoanalytic theory is concerned with the emotional development of the individual. It is an applied science, an extension of physiology, which expands the boundaries of social science and conducts the study of human nature. Winnicott argues that psychoanalysis is about the unconscious, about the life hidden deep within the individual’s heart, rooted in the real and the imagined life of the individual’s early childhood (2). And psychoanalysis is not just about interpreting the repressed unconscious, it also provides a professional setting for building the trust in which analytical work can take place. Psychoanalysis has greatly influenced the way people view life.

### 2.2. The purpose of psychoanalysis

Different from traditional psychoanalysts, Winnicott puts forward bluntly that the purpose of psychoanalysis is “keeping alive; keeping well; keeping awake” (4, p. 166), to be himself and express himself. The success of the psychoanalyst means that he can survive a psychoanalytic session after experiencing the patient’s use of the object and ending the analysis smoothly. Winnicott points out that the analyst conducts psychoanalysis because the patient needs him to do it. Before beginning the psychoanalysis, the analyst should continuously make a personal and social diagnosis of the patient. Winnicott believes that the diagnosis of the patient cannot be separated from the social position of the individual, and the psychoanalysis is only initiated when it is confirmed that the individual needs psychoanalysis in his environment. In general, psychoanalysis is for those who want it, need it, and can afford it (4).

## 3. Classification of diseases caused by psychological reasons

Winnicott points out that disorders caused by psychological rather than physical causes are due to the immaturity of the individual, that is, immaturity in the emotional growth of the individual. It represents the obstacle in the process of the individual’s emotional development, and the direct goal of psychoanalysis is to remove this obstacle, so that the individual’s development can continue smoothly. Winnicott divides the psychological disorders caused by the immaturity of the individual into three categories (5).

### 3.1. Psychoneurosis

The first category is psychoneurosis (5). Individuals with these disorders are well cared for in the early stages, but as they develop, they encounter some of the inherent difficulties in life, but fail to overcome them. For example, normal children may experience some severe anxiety simply because of interpersonal conflicts which are inherent parts of life and the management of instincts. In normal life, the individual should be able to control his instincts and not develop a disease due to such difficulties. Winnicott classifies depression into this category.

### 3.2. Disorders as psychosis

The second category is psychosis, resulting from failure in infant nurture (5). Such patients suffer from disturbances in the development of their personality structures because of inappropriate care given in very early infancy. This care deficit may lead to psychosis in infancy or childhood. Winnicott argues that such disorders may manifest themselves under a variety of stress, such as the stress of trauma, and the stress of adolescence.

### 3.3. Disorders due to deprivation of good environment

The third category is a mental illness caused by deprivation of a good growth environment (5). The care that these individuals received in the early stages was good enough, but at some point, their good upbringing environment was lost. The self-sustaining existence of the individual who should have been raised in a good enough environment was interrupted and converted into a response to environmental failure, and this state is called “deprivation.” At this point, the individual’s self-organization develops to a considerable extent, but fails before becoming independent. Deprivation possibly leads individuals to develop an antisocial tendency which may manifest into juvenile delinquency. These individuals may steal or wreak to force the environment to notice or act upon them (5).

## 4. Treatment methods for mental illness

### 4.1. Treatment of psychoneurosis

Winnicott believes that the first category psycho-neurosis, if treatment is needed, should be provided with psychoanalysis, of which Freud’s classical psychoanalytic setting is more effective (5). In a professional setting, patients will present their past experiences and manifest their inner reality, exposing themselves to the fantasy which is their ever-changing relationship with the analyst. Through psychoanalysis, the repressed unconscious becomes conscious, and once the process of the analysis begins, the analyst obtains the patient’s unconscious cooperation, and it also determines the length of the average treatment.

An important characteristic of such patients is the desire for self-consciousness, and the main work of psychoanalysis is to make the patient’s subconscious mind conscious. For these people, analysis can make them more aware of the contents of the analysis and more tolerant of unconscious feelings. In psychotherapy, the patient cannot reserve areas against the analyst, and if a patient keeps part of himself completely defended, it will avoid the instinct to trust in the treatment process, which is crucial to the effectiveness of the therapy. The material analyzed in psychoanalytic therapy is not the patient’s delusions, but dreams, imaginations, and ideas expressed in symbolic form (5). As the treatment process goes on, the patient’s unconscious cooperation continues to develop, and those symbolic forms will be interpreted, thereby achieving the therapeutic effect.

### 4.2. Treatment of disorders such as psychosis

Winnicott points out that the second category of disorders psychosis are ascribed to a failure in nurture, which distorts the early stages of an individual’s emotional development due to defective infancy care. What can be seen from the patient is a failure of the structure of the personal self, the structure of the individual ego, and the ability of the self for relating to objects that are of the environment. These various failures may constitute the clinical manifestations of psychosis or subconsciousness, interfering with the patient’s normal life. Such patients should be treated and cared for from a psychiatric perspective, rather than by physical therapy and drugs.

If the illness of this category is caused by failures in early nurture, the analyst needs to provide the patient with a holding environment so that he can feel the dependence experience in the early care stage and regain his early subjective sense of omnipotence. The analyst, the family that continues to care for the patient, or the society provides the patient with a holding environment that allows the patient to regress to the state of dependence. If the regression needs can be met, and the holding environment can replace the failure environment, the patient will be able to experience the success of the holding environment that promotes the patient’s emotional development, experience self-increasing independence, and feel satisfied. And the emotional experience of failure will be replaced, that is, regression brings the patient an opportunity for rectification, in which the emotional experience of failure in infancy can be corrected (6).

### 4.3. Treatment of disorders due to deprivation of a good environment

According to Winnicott, for the third category of illness caused by deprivation of a good environment, treatment needs to be adapted to the fact that the patient feels the “deprivation,” and full attention and recognition should be given. To deal with the patient with antisocial tendencies triggered by deprivation, the analyst should try to tolerate the patient’s antisocial tendencies, as they are evidence that the patient is still hopeful, which means that they are sending out S.O.S signals, calling on the social environment to acknowledge the wrongs they have committed, and calling on the society to recreate a good environment for the patient (2).

During treatment, the analyst usually can find out therapeutic clues and solutions through serious digging done in the early stages of the patient’s antisocial career. Winnicott suggests that the analyst should focus on antisocial tendencies in patients whose families are intact and relatively normal. In such cases, the analyst is likely to know the events of deprivation that alter the entire trajectory of the patient’s emotional development. Psychotherapy for a patient with antisocial tendencies will only work if the patient is near the beginning of his antisocial career and before his delinquent skills have behaved more likely been established. Because it is only at this early stage that the patient will know that he is a patient and will want to know the roots of the disturbance. Winnicott points out that, in many cases, treatment can be effective if it is introduced at an earlier stage. In particular, the mild antisocial tendencies of some children can be cured by the good family life of parents and children. For those cases where antisocial tendencies have built up into a stabilized juvenile delinquency, specialized environments need to be provided. One is the holding environment in which children are socialized; the other is the environment that isolates children and keeps them away from society, the kind of institutions like juvenile delinquents detention centers (5).

### 4.4. Choice of treatment method

Winnicott believes that there are many varieties of psychotherapy, and the type used should depend not on the practitioner’s views, but on the need of the patient. For example, the analysis of psychosis and neurosis are inherently different. The former requires the analyst to be able to endure the regression behaviors of the patient in reality, while the latter requires the analyst not only to be able to tolerate the patient’s strange thoughts and feel the patient’s love and hate contradictions, but also to interpret his understanding of the patient through appropriate expressions (7). An appropriate and well-timed verbal interpretation in psychotherapy will give the non-psychotic patient a feeling of being physically held, a more authentic feeling than a real hugging or caring behavior that the patient has needed in the past. Winnicott notes that the difference between analyzing children and analyzing adults is that during treatment, children behave in a playing way, while adults behave more likely in their own life than in the analysis. He believes that patients, no matter how old they are, prefer the analyst to treat them in the simplest way (7).

Winnicott holds that one essential of psychotherapy is that it should not be mixed up with other treatments. Because it will change the whole clinical process. The patient may either fear or secretly expect the effects of other treatments (or both), and the analyst may never find out the patient’s real problem.

## 5. Professional attitudes of the psychoanalyst

Winnicott puts forward that the professional attitude of the psychoanalyst is extremely essential in therapy. The professional attitude of psychoanalysis is similarly symbolic, because it assumes the distance between the analyst and the patient, which is located in the gap between the subjective object and the object of the objective perception (2).

### 5.1. Establishing trust

In therapy, the psychoanalyst should be objective. The analyst accepts and is influenced by the patient’s love and hate, but he does not provoke the patient’s love and hate, nor does he hope to get emotional satisfaction in a treatment relationship (2). He has to maintain a professional engagement as well as not too much tight tension. Within his career, the analyst is a person who is deeply involved in various emotional feelings yet still maintains the ability to separate, because he knows that he has no responsibility for his client’s illness, and he also knows that his ability to save the patient’s crisis is limited to some degree.

At the beginning of psychoanalytic treatment, the analyst should be careful not to be too clever. In the first interviews, the patient usually brings up all his believes and all his suspicion. These extremes must be allowed to express faithfully. If the analyst does too much at the very beginning, the patient will either run away or develop obedience out of fear.

Winnicott notes that a psychoanalytic treatment depends on the patient’s ability to trust others and also depends on whether the analyst can prove himself worthy of trust. To build mutual trust, there is a long preparation period between the analyst and the patient before starting the treatment.

### 5.2. Adapting to the needs of the patient

During the therapy, the psychoanalyst needs to understand the patient’s feelings and try to use various methods to meet the patient’s needs. Usually, the patient does not know what the analyst does well, but when things go wrong, the negative role of the analysts is perceived. Because precisely when the analyst fails, the patient responds to the failure, and the continuity of his self-persistence is interrupted. Therefore, the analyst must avoid leaving the patient shortly after starting an analytic treatment, which is a test of the vulnerability of the patient’s ego, because the patient is not ready to deal with the analyst’s departure (3). The departure of the analyst is like one of the various types of environmental failures that constitute the individual’s early fundamental deficiencies in parenting, which can lead to the annihilation of the individual whose self-sustaining existence has been interrupted. Because the individual is not yet able to use projection to explain this environmental failure, and the individual has not yet reached the stage of the self-structure which is required to use the projection mechanism (6).

When regression occurs in the therapeutic process, the patient (no matter what age) must eventually be able to achieve unconscious awareness of the care in the environment and dependence on himself, which means that the analyst can adapt well enough to the needs of the patient. In the recovery process, the analyst needs to play two roles- the worst and the best in the patient’s imagination (an idealized mother role, giving the patient perfect care) (7). With the recognition of the idealized and the very bad role played by the analyst, the patient gradually accepts the good and the bad, the hopeless and the hopeful, the unreal and the real part of the self.

Winnicott claims that the foundation of the individual’s mental health is actively laid during the infancy when the mother can perform her duties well enough (6). The emotional mechanism of the mother–child bond is the basic principle of the psychoanalyst’s work, which is the working basis for a psychoanalyst in treating children whose early mothering is not good enough (who cannot perform their duties of caring for babies well) or was interrupted (8). During the therapeutic work, the analyst is involved with the patient over and over again. The patient and the analyst will experience a vulnerable phase due to the involvement. Like the mother, the analyst will temporarily identify with the patients who depend on him. The analyst observes the shedding of the patient’s false self, and sees the new beginning of a true self. Because, like the mother and her baby, the analyst can give support to the ego of the patient. If all goes well, the birth of the patient’s new self can be seen, and the patient can begin to have an independent life, in which he can organize his own defenses against the instinctive impulse and experience the realities and difficulties of the life itself. All an analyst can do in treatment is to imitate the natural process a mother does to her baby, to fully meet the patient’s needs (8).

## 6. Unique concepts of Winnicott’s treatment views

Winnicott has put forward many innovative theories which significantly contribute a lot to the study of the healthy development of the individual’s mental growth. His classic representative concepts, especially the ideas of “good-enough mother,” “facilitating environment,” “true self and false self” and “transitional object and transitional phenomena,” have laid the foundation of his psychoanalytical treatment views, which provide positive perspective for the development of the individual, as well as give a worthwhile reference for psychoanalysts and children parenting.

### 6.1. A good-enough mother and the true self

“Good-enough mother” is his trademark language among his marvelous theories. “True self” and “false self” are the core concepts of Winnicott’s outlook on the individual’s growth. Winnicott links the individual’s development of the self with good interaction between the mother and the child during his early growth. Winnicott sees the individual’s experience in the first few months of his infancy as the key to the development of his personality. He believes that a good-enough mother can allow the child to develop his true self. If the mother is not good enough, then the child may produce a compliant false self, and the true self will presumably hide behind the false self.

Winnicott emphasizes the important role of the environment in the process of self-formation of the individual. He asserts that no matter whether the environment is suitable or not, it will surely affect the individual’s self-development. Winnicott points out that the infant’s mother is his first environment. “There is no such thing as a baby” (9, p. 88), he says, when we see a baby, we also see the mother who looks after it. He refers to a “good-enough mother” as the “facilitating environment.” According to him, “facilitating” means adaptation to the basic needs of the infant. A good-enough mother will timely meet the needs of the infant and provide good support for his growth. Having a facilitating environment, the infant’s self will be successfully integrated during the development, having the ability to establish self-consciousness and face the potential difficulties in the external world.

Winnicott states that, at the very beginning, a good-enough mother is in her “primary maternal preoccupation” (6). During the early stages of pregnancy and birth, a good-enough mother will withdraw from her own interests and highly focus on the baby’s wishes. By feeding the baby when he is hungry and comforting the baby when he cries, the mother can meet the baby’s needs with almost perfect accuracy, thus giving the baby the illusion that things are created by his own wishes. In this way, the baby gradually integrates from his omnipotent fantasy to produce a true self, with a sense of reality. If the mother is not good enough, the needs of the baby will not be well satisfied. Instead, she may force the baby to accept her own will. With the needs of the baby being missed again and again, the baby will not feel the subjective sense of the world, but experience the conflict between reality and fantasy. While realizing that it is an external world that he must immediately compromise with and adapt to in his early infancy, he will be forced to develop a defense mechanism that shows compliance. And this phenomenon of submission is the earliest stage of the false self.

Winnicott regards that, as the individual grows, a good-enough mother will gradually turn to her own life from the initial state of primary maternal preoccupation and fail to give timely feedback to her babies’ needs. Sensing the changes of the mother, the baby comes to recognize that the world around him is not run by his own desires, and begins to find himself involved with the environment. With feeling the interaction and dependence on the environment, the baby gradually builds his self-consciousness. While his consciousness of “self” and “object” is emerging clearer, the separation of “self” and “object” naturally ensues. And the baby will also gradually acquire the ability to face the external reality. The key for a good-enough mother to be in a facilitating environment is to be there when the child needs her and to back off voluntarily when the child no longer needs her. The significance of a facilitating environment is to adapt to the growth of the child and encourage him to become independent.

Winnicott believes that the early supporting environment the mother provides determines the result of the development of the individual’s personality. The omnipotent almighty subjective feelings a good-enough mother brought the baby can help the individual to build a sense of reality, a feeling of psychological security, and lay a good psychological foundation for the healthy development of the individual’s true self. Winnicott asserts that in the earliest stages of life, a good enough environmental supply enables the infant to begin to exist, to have experiences, to build an individual self, to harness instincts, and to face the difficulties inherent in life. He also points out that if a child does not have a good environmental supply early in life, then his self will not develop, he will lack a sense of reality, and he may experience all sorts of psychological problems as he grows up. Even if the child caretaker strives hard to make up for it later in the nurturing process, it will not necessarily change the distortion suffered by the early self-development (4).

In his treatment theoretical work, Winnicott emphasizes the overwhelming importance of the facilitating environment especially a good-enough mother to the foundation of the true self. Just as Winnicott says, “It is an essential part of my theory that the true self does not become a living reality except as a result of the mother’s repeated success in meeting the infant’s spontaneous gesture or sensory hallucination.” (4, p. 145) For Winnicott, an analyst can try to imitate a good-enough mother by providing the patient with a facilitating environment during the process of treatment so that he can get the ability to return to the state of absolute dependence to face the original traumatic situation, modifying his previous traumatic experiences and resulting in the emergence of the true self. Winnicott states that the effectiveness of regression during the treatment relies on whether the individual can achieve independence which depends on the individual’s competence to trust the analyst. And the analyst needs to prove that he can be trusted as a good-enough mother. It is obvious that, in terms of Winnicott’s theories, the facilitating environment such as a good-enough mother is the essential prerequisite for both the emergence of the true self and the recovery of the false self.

### 6.2. Transitional object and transitional phenomena

“Transitional object” and “transitional phenomena” are important concepts put forward by Winnicott. He believes that the transitional object is the first Not-Me possession of children. Once the infant has subjective feelings and is separated from the mother, he will create a Not-Me possession of his own, which is called the “transitional object” (6). The first Not-Me possession of a child is often a blanket or a small toy. The smell or texture of the transitional object is associated with the mother to some extent, which can take the place of the mother to soothe the baby’s emotions. The parents know the value of the transitional object. They let it get dirty or smelly without cleaning due to being afraid to break the continuity in the infant’s experience, which may destroy the meaning of the object to the infant (6). Through the transitional object, the child can improve his ability to withstand separation and self-reliance. It is between the internal object and the external object, which is the effective connection between the mother and the baby. Winnicott claims “The object is a symbol of the union of the baby and the mother (or part of the mother) …The use of an object symbolizes the union of two now separate things, baby and mother, at the point in time and space of the initiation of their state of separateness” (10, p. 4). Winnicott calls all these things involving transitional objects transitional phenomena (6). Transitional phenomena refer to the process of using the object as a means of making the transition from an external to an internal experience. This process involves a series of stages, such as the infant’s initial attachment to the object, the development of a sense of play with the object, and the eventual relinquishing of the object as the child internalizes its comforting qualities.

**Figure 1:**
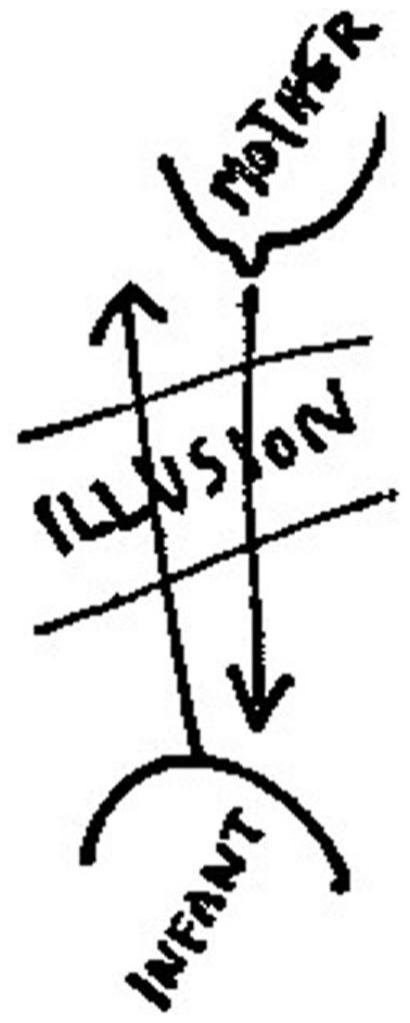
The use of illusion.

**Figure 2:**
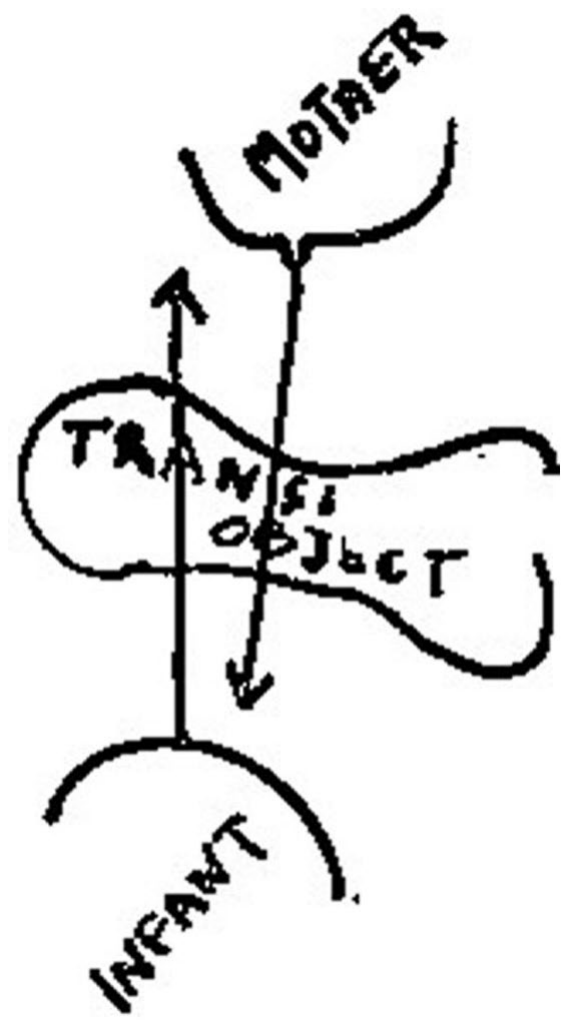
The use of transitional object.

Winnicott associates transitional objects and transitional phenomena with the realm of illusion, which plays a fundamental role in the infant’s initiation of experience. He claims that the transitional phenomena signify the initial phases of the use of illusion, which is crucial for the individual to establish a sense of relationship with an external object. Without the illusion, there would be no meaning in such a relationship (6). Furthermore, Winnicott emphasizes that the ability to use transitional objects depends on a good enough mother. Winnicott illustrates his ideas by the following two figures, Figures 1, 2 (6). Figure 1 states that a good-enough mother’s adaptation to the infant’s needs gives the infant the illusion that he can create what he needs. Psychologically, the infant takes the mother’s breast as part of himself, and the mother gives him milk is also part of himself. That is to say, this early stage in the development of the individual becomes possible by the mother’s ability to adapt to the infant’s needs, enabling the infant to have the illusion that what he creates does exist. In psychology, the interchange between mother and infant is based on an illusion (6). As the infant grows, the mother’s main task is to provide disillusionment, which means the task of weaning. If things go well, weaning is the process of gradual disillusionment, the stage of frustration for the infant. The complex reaction of weaning is actually the illusion-disillusionment process that the infant undergoes. In experiencing the frustrations, the infant forms a conception of external reality. Figure 2 gives a shape of the area of illusion, to illustrate what Winnicott considers to be the main function of the transitional object and the transitional phenomena. It illustrates how the region of the illusion is transformed into a definite form of a transitional object, which illustrates the main mechanism for the formation of transition objects and transition phenomena as the individual develops. Winnicott points out that, the transitional object and the transitional phenomena prove to be very important for each human being (6). He also argues that the use of the transitional object and the process of transitional phenomena are not limited to childhood but could also occur in adulthood, particularly in the context of psychotherapy.

Winnicott believes that there is a third spiritual world between the inner world and the outer world, namely the intermediate area, or the potential space, or the transitional space. The external world is the physical reality we live in. The internal world is the realm of our internalized object relations. And the third world, also known as the intermediate area, potential space, or transitional space, is the middle area of experience, which lies between fantasy and reality. It enables reality from fantasy and omnipotence. Winnicott claims that this intermediate area is closely related to the generation of plays and symbols, and belongs to the category of plays, creativity, fantasy, imagination, and illusion. Children’s playing is a transitional space, from which children develop interpersonal skills (11). He says “The intermediate area to which I am referring is the area that is allowed to the infant between primary creativity and objective perception based on reality-testing” (6).

The transitional space allows for the child’s imagination and creativity to flourish. This space is not only a physical space, but also a psychological and emotional one and it provides the child with the freedom to explore and develop his sense of the self. In the transitional space, the child can better connect the subjective world with the real world with the help of the transitional object, promoting the development of the infant’s thinking. For example, to meet his psychological needs, the infant will treat the small toys around him as what he wants to see, and gradually get used to being separated from his mother. In this way, the infant gradually accepts the real world with the help of his own created fantasy and develops the ability to interact with the real world, and no longer relies on the mother completely. Winnicott believes that infants and toddlers spend most of their time in transitional space, in which the transitional object, as the inner mother, can take the place of the mother at some point. For example, some babies like to suck their thumbs. At this time, the thumbs can take the place of the mother to comfort the babies and relieve their psychological insecurity. The transitional space plays a positive role in promoting the healthy growth of children. Winnicott states that, in favorable circumstances, this potential space becomes filled with the products of the baby’s own creative imagination (10).

Winnicott’s views about the transitional object, transitional phenomena, and potential space have important implications for psychotherapy. He sees the creation of a potential space as a crucial aspect of therapy. It is important to provide the patient with a safe and supportive environment in which he could use the transitional object, such as play or drawing, to explore his inner world and emotions. He argues that this process is essential for the patient to achieve a lasting sense of emotional well-being, as he internalized the experience of security and comfort that had been provided by the therapist. It can help the patient develop his true self.

In addition, Winnicott relates the transitional objects and the transitional space to a good-enough mother or a facilitating environment, and he claims:

In infancy this intermediate area is necessary for the initiation of a relationship between the child and the world, and is made possible by good enough mothering at the early critical phase. Essential to all this is continuity (in time) of the external emotional environment and particular elements in the physical environment such as the transitional object or objects (6, p. 241).

Therefore, according to Winnicott, it is necessary to make good use of the transition object to constantly enrich the experience of the infant’s transitional space, in his early development process. It can help the infant to establish his own transitional space, create and transform the external real world through the transitional space, so that the infant can gradually connect with the real world and build effective defense mechanisms in the internal world. It is conducive to improving the infant’s symbolic thinking ability, enhancing the infant’s self-experience, and establishing healthy and developing object relations. Winnicott points out that playing is helpful in promoting the unity and comprehensive integration of personality. In the early growth of the individual, through the good experience of transitional objects such as playing, the transitional space will build a bridge for the healthy development of the child’s real self (6).

In conclusion, Winnicott’s marvelous treatment views, such as the true self and false self, the transitional object, and the transitional phenomena, continue to be highly influential in treatment approaches to contemporary psychotherapy.

## 7. Related research areas and hotspots

To figure out the research status and hotspots of Winnicott’s psychological treatment views in international research circles, we use CiteSpace software to conduct a clustering analysis based on the database of WoS Core Collection. We intend to present a macroscopic and panoramic study of Winnicott’s related theories, by summarizing the contents of the selected literature and exploring the hot topics, evolution characteristics, and future development trends of research on Winnicott with multiple dimensions such as figure and table.

In the advanced search in WoS Core Collection, we set the search query as: TS = (Winnicott AND psychoanalysis) OR TS = (Winnicott AND mentality) OR TS = (Winnicott AND emotion) OR TS = (Winnicott AND treatment). A total of 416 records were retrieved. The retrieval date was Feb. 15, 2023. After deleting some book reviews and irrelevant documents, we got 365 items. In this study, only original articles, proceeding papers, and review articles were included. We excluded editorial material, book reviews, biographical-items, book chapters, and duplicated documents. We imported all records into CiteSpace to cluster the keywords. Then, this study mainly focuses on analyzing the visualization results of annual publications, most productive countries, institutions, and authors, as well as hot keywords and keyword clusters. Furthermore, it summarizes the categories and the hotspots of existing research.

**Figure 3:**
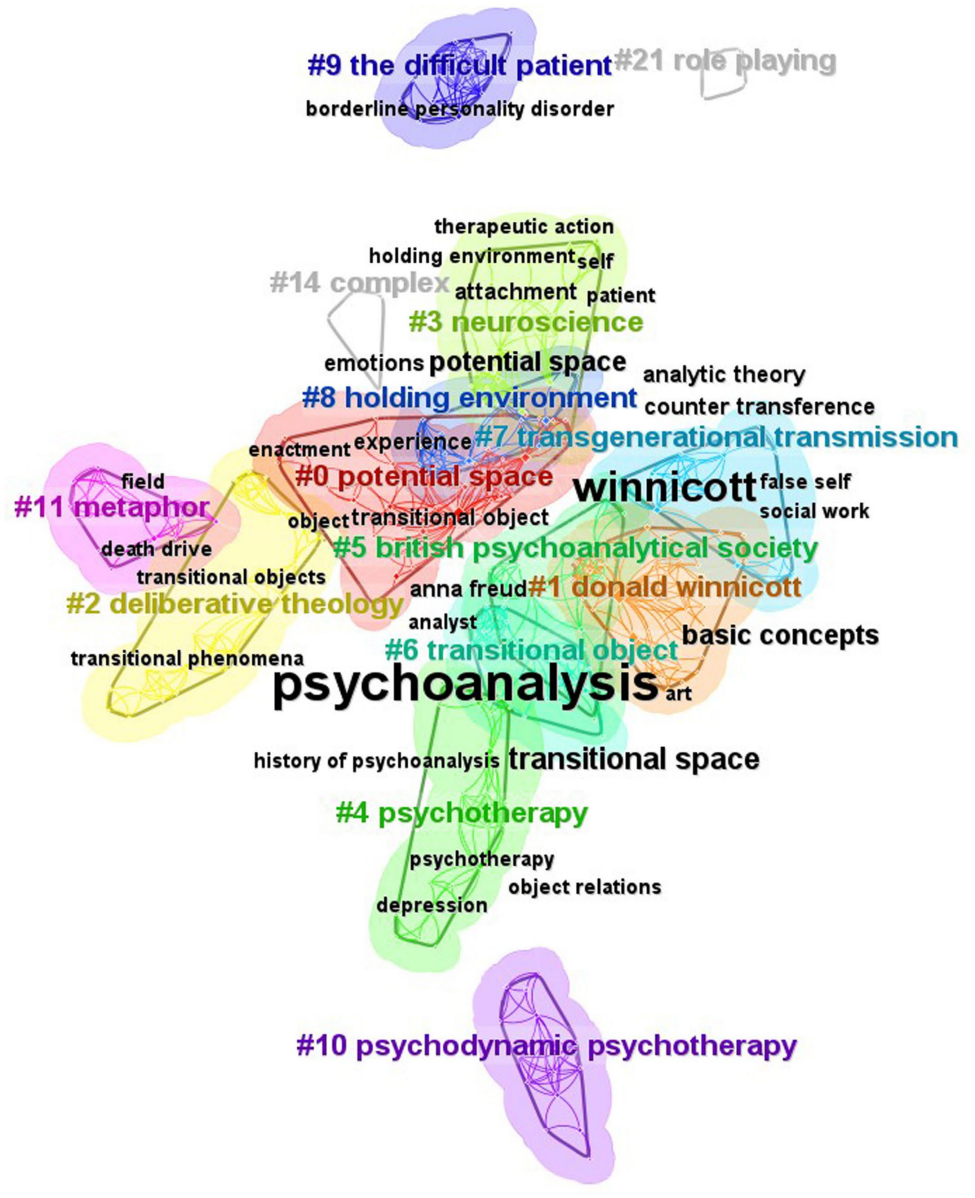
The keyword clusters.

The results, received through the log-likelihood ratio (LLR) algorithm, show the 14 largest keywords clusters in Figure 3. According to CiteSpace, the Modularity Q > 0.3 means that the divided community structure is significant; the Weighted Mean Silhouette S > 0.5 indicates that clustering is reasonable, and S > 0.7 indicates that clustering is highly efficient and convincing. In this study, the Modularity Q is 0.8164, which means the network community structure is significant and with good clustering effect; while the Weighted Mean Silhouette S is 0.9568, not only much greater than the critical value 0.5, but also greater than 0.7, indicating that the clustering results are quite convincing. Therefore, the remarkable structure and good effect of clustering results of this study can effectively help to find out the overall characteristics and development trend of research on Winnicott’s psychological treatment views.

### 7.1. Annual publications

To some extent, the time of publication and the distribution of the annual publication volume directly reflect the research trend and the development speed in the research field. From the statistics in CiteSpace, the international study of Winnicott’s psychological treatment views began in 1978. Supplementary Table S1 illustrates the annual publication trend from 1978 to 2023.

As can be seen from Supplementary Table S1, except for only sporadic documents in the first two decades, the number of documents of research on Winnicott has increased year by year, especially after 2004. This shows that Winnicott’s theories have attracted more and more attention from scholars, and the popularity of research is gradually increasing.

In terms of the annual publications trend chart (Supplementary Table S1), researches on Winnicott’s theories can be roughly divided into four periods: the first period is the years of 1978–1991, the number of publications in this phase is approximately null and with slow growth; the second period is years of 1992–2004, while the average annual number of publications is 6, nearly 6 times of the first period; the third period is years of 2005–2016, when the growth rate of the number of documents is significantly faster than the previous period, with an average annual volume of 12 articles, nearly 2 times of the second period; the last period is years of 2018–2023, and the number of documents in this period has increased significantly based on the previous period. Despite of temporary small decrease in 2020 and the incomplete statistics in 2023, the average annual number of documents in this period has reached 28, 2.3 times of the third period and 28 times of the first period. It can be concluded that research on Winnicott’s theories has entered a stage of steady rapid development and in-depth research, and has received obvious attention from the academic circle.

### 7.2. Country analysis

We use CiteSpace to analyze the country-level collaboration. In our study, there are a total of 32 countries involved in the research of Winnicott. As shown in Table 1, USA ranks the first with 102 publications, far ahead of the second place England with 42 publications. Germany ranks the third with 20 publications. All of these countries have deep research into Winnicott.

**Table 1:** Top 10 most productive countries.

No.	Publications	Country	No.	Publications	Country
1	102	USA	6	16	Brazil
2	42	England	7	8	Italy
3	20	Germany	8	6	Canada
4	18	France	9	5	Sweden
5	17	Israel	10	5	Australia

**Figure 4:**
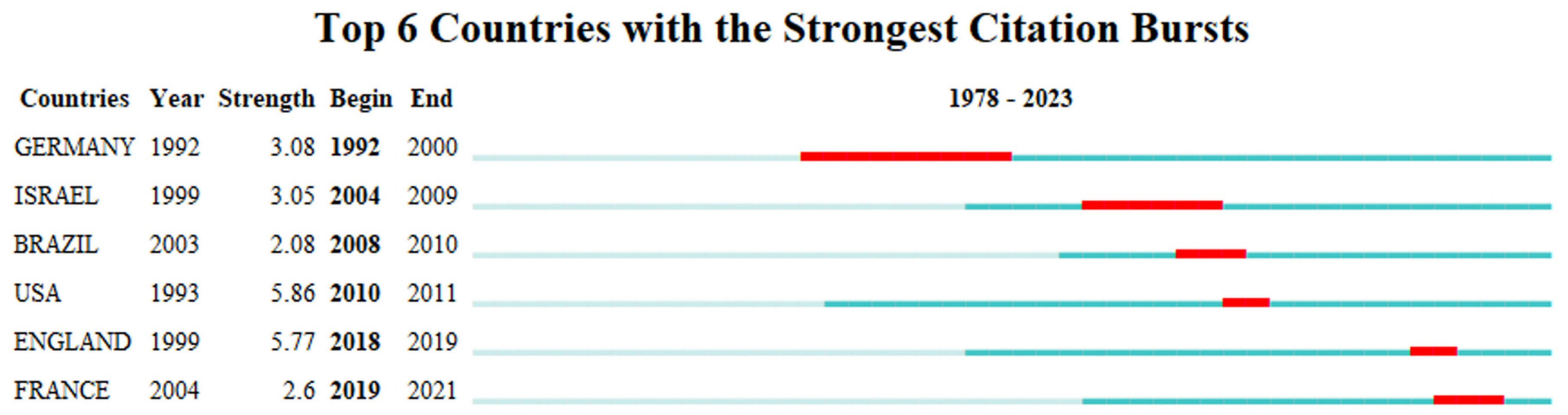
Top 6 Countries with the Strongest Citation Bursts.

As shown in Figure 4, it is interesting to notice that Germany had a strong citation burst from 1992 to 2000, which means that during that time Germany had profound research into this topic. From 2004 to 2009, it was Israel that had a strong citation burst. But Israel started this research at the year of 1999. The USA started the research at the year of 1993, just 1 year after Germany. It had the strongest citation burst from 2010 to 2011, which only lasted for 1 year.

### 7.3. Institution analysis

Among the top 10 most productive institutions (Table 2), 5 (New York University, Harvard University, Psychoanalytic Center of California, Smith College, and Northwestern University) are in the USA, which is responding to the result that the USA is the most productive country. 2 (University College London, and British Psychoanalytical Society) are in the UK. 2 (Tel Aviv University, and Hebrew University of Jerusalem) are in Israel. Only 1 (Aix Marseille University) is in France. New York University ranks the first with 10 publications. Harvard University follows, with 6 publications. Besides universities and colleges, research centers are also interested in this research, such as the Psychoanalytic Center of California and the British Psychoanalytical Society.

**Table 2:** Top 10 most productive institutions.

No.	Publications	Institutions	Country
1	10	New York University	USA
2	6	Harvard University	USA
3	6	University College London	UK
4	5	Tel Aviv University	Israel
5	5	Psychoanalytic Center of California	USA
6	4	Smith College	USA
7	4	Aix Marseille University	France
8	3	British Psychoanalytical Society	UK
9	3	Northwestern University	USA
10	3	Hebrew University of Jerusalem	Israel

### 7.4. Author analysis

The top 10 most productive authors are shown in Table 3. Among them, Ofra Eshel and Marie Lenormand rank the first with 5 publications. Ofra Eshel (12) mainly focuses on the radical departure of Bion’s and Winnicott’s clinical ideas from traditional psychoanalytic work, particularly emphasizing the depths of early breakdown and regression in the treatment of disturbed patients. Marie Lenormand (13) reevaluates the framework of child psychoanalysis and explores the heterogenous ensemble of discursive and non-discursive factors that contribute to the analytical process, by focusing on Winnicott’s case of The Piggle.

**Table 3:** Top 10 most productive authors.

No.	Publications	Author	No.	Publications	Author
1	5	Eshel, Ofra	6	3	Kirshner, Lewis A
2	5	Lenormand, Marie	7	3	Bonaminio, Vincenzo
3	4	Cooper, Steven H	8	2	Gerard, Nathan
4	4	Aguayo, Joseph	9	2	Fabozzi, Paolo
5	3	Groarke, Steven	10	2	Gauthier, Martin

### 7.5. Keywords clusters

Through the Keywords module of CiteSpace software, the visualization results of keywords frequency and keyword clustering of Winnicott’s psychological treatment views are conducive to analyzing the hot topics in this field.

Figure 3 demonstrates the 14 largest keyword clusters. It includes cluster #0 potential space, cluster #1 donald winnicott, cluster #2 deliberative theology, cluster #3 neuroscience, cluster #4 psychotherapy, cluster #5 British psychoanalytical society, cluster #6 transitional object, cluster #7 transgenerational transmission, cluster #8 holding environment, cluster #9 the difficult patient, cluster #10 psychodynamic psychotherapy, cluster #11 metaphor, cluster #14 complex, and cluster #21 role playing. The smaller the number of the cluster ID, the more important is the cluster.

Table 4 reveals the main keywords of the 14 keyword clusters in Figure 3.

**Table 4:** Main keywords of 14 keyword clusters.

Clusters ID	Main keywords
#0 Potential space	Potential space; trauma; intersubjectivity; rorschach; dichotomy
#1 Donald Winnicott	Donald winnicott; transitional space; the future of an illusion; compassion; social work
#2 Deliberative theology	Deliberative theology; transitional objects; healthcare students; content analysis; internalized object representation
#3 Neuroscience	Neuroscience; primitive experience; reciprocal interactive intervention; symbolic order; infant as subject
#4 Psychotherapy	Psychotherapy; stolorow; borderline patients; improvisation; rupture
#5 British psychoanalytical society	British psychoanalytical society; history of psychoanalysis; w. r. bion; infant development; melanie klein
#6 Transitional object	Transitional object; bilateral hippocampal injury; objectual practices; intersubjective; symbol
#7 Transgenerational transmission	Transgenerational transmission; el vinculo; tomkins; emergent organization; affect
#8 Holding environment	Holding environment; dream interpretation; situation; boundary violations; theory of neuronal group selection
#9 The difficult patient	The difficult patient; incohesion; mass psychology; valence; the fourth basic assumption
#10 Psychodynamic psychotherapy	Psychodynamic psychotherapy; addiction; cocaine; winnicott; psychoanalysis
#11 Metaphor	Metaphor; death drive; deobjectification; andre green; work of the negative
#14 Complex	Complex; modell; ogden’s dialectical space; archetype; transcendent function
#21 Role playing	Role playing; analytic field; analytic setting; ogden; origami

In order to explicitly show the major research areas, we take the 5 largest clusters among the 14 keyword clusters into a deep analysis, including cluster #0, cluster #1, cluster #2, cluster #3, and cluster #4. Detailed information about the 5 largest keyword clusters is shown in Table 5.

**Table 5:** The five largest keyword clusters.

Cluster ID	Size	Silhouette	Mean (Year)	Top Terms (LSI)	Top Terms (log-likelihood ratio, *p*-level)	Terms (mutual information)
0	47	0.934	2012	Potential space; self creation; authentic self expression; new self organization; Winnicott | psychoanalysis; intersubjectivity; mutual recognition; work; narcissism	Potential space (12.1, 0.001); trauma (6.01, 0.05); intersubjectivity (3.3, 0.1); Rorschach (2.99, 0.1); dichotomy (2.99, 0.1)	rorschach (0.8); dichotomy (0.8); eating disorders (0.8); metaphysics (0.8); adolescent psychoanalysis (0.8)
1	42	0.936	2012	Transitional space; infant observation; scenic understanding; cultural analysis; patient-analyst interconnectedness | donald winnicott; bruno bettelheim; cognitive disability; sigmund freud; patient-analyst interconnectedness	Donald winnicott (6.01, 0.05); transitional space (6.01, 0.05); the future of an illusion (3.34, 0.1); compassion (3.34, 0.1); social work (3.34, 0.1)	The future of an illusion (0.64); compassion (0.64); social work (0.64); scene (0.64); suffering versus feeling pain (0.64)
2	29	0.983	2012	Deliberative theology; transitional objects; internalized object representation; spiritual issues; object concept | content analysis; healthcare students; deliberative theology; transitional objects; internalized object representation	Deliberative theology (6.91, 0.01); transitional objects (6.91, 0.01); healthcare students (6.91, 0.01); content analysis (6.91, 0.01); internalized object representation (6.91, 0.01)	Deliberative theology (0.06); transitional objects (0.06); healthcare students (0.06); content analysis (0.06); internalized object representation (0.06)
3	25	0.969	2011	Primitive experience; therapeutic regression; analytic technique; maternal metaphor; noetic feeling | reciprocal interactive intervention; therapeutic action; mirror neurons; noetic feeling; maternal metaphor	Neuroscience (5.48, 0.05); primitive experience (5.48, 0.05); reciprocal interactive intervention (5.48, 0.05); symbolic order (5.48, 0.05); infant as subject (5.48, 0.05)	Neuroscience (0.16); primitive experience (0.16); reciprocal interactive intervention (0.16); symbolic order (0.16); infant as subject (0.16)
4	22	0.967	2006	Severity; borderline patients; depression; psychotherapy; treatment duration | object relations; severity; treatment duration; borderline patients; depression	Psychotherapy (5.75, 0.05); stolorow (4.66, 0.05); borderline patients (4.66, 0.05); improvisation (4.66, 0.05); rupture (4.66, 0.05)	Stolorow (0.27); borderline patients (0.27); improvisation (0.27); rupture (0.27); music (0.27)

From Table 5, we can see the largest cluster #0 potential space contains 47 documents. The silhouette is 0.934. The keywords belonging to this cluster are potential space, trauma, intersubjectivity, Rorschach, and dichotomy.

The second largest cluster #1 donald winnicott contains 42 documents. The silhouette is 0.936. The related keywords are Donald Winnicott, transitional space the future of an illusion, compassion, and social work.

The third largest cluster #2 deliberative theology contains 29 documents. The silhouette is 0.983. The keywords in this cluster are deliberative theology, transitional objects, healthcare students, content analysis, and internalized object representation.

The fourth largest cluster #3 neuroscience contains 25 documents. The silhouette is 0.969. The keywords are neuroscience, primitive experience, reciprocal interactive intervention, symbolic order, and infant as the subject.

The fifth largest cluster #4 psychotherapy contains 22 documents. The silhouette is 0.967. The keywords are psychotherapy, stolorow, borderline patients, improvisation, and rupture.

The Silhouette of the 5 largest clusters are all much greater than 0.7, and the keywords of Top Terms such as potential space, psychoanalysis, infant observation, Sigmund Freud, and transitional object are convincingly showing the hot topics in the field of research about Winnicott.

According to the top 30 hot keywords of high frequency generated by CiteSpace software in Table 6, it can be seen that the keyword with the highest frequency is “psychoanalysis” with 36 times and the second is “Winnicott” with 17 times. This reflects that “psychoanalysis” and “Winnicott” are the basic elements and key support for the practice of this field. In CiteSpace, centrality scores are normalized to the unit interval of [0, 1]. A node of high centrality is usually one that connects two or more large groups of nodes with the node itself in between (14). Other keywords with a high degree of centrality are “potential space,” “transitional space,” “transitional object” and “object,” together with notable keywords “emotion,” “analytic theory” and “counter transference,” which are also the key nodes in the network structure of Figure 3. It implies that Winnicott’s theory of psychoanalysis such as concepts of transitional space or object has aroused researchers’ great interest in recent years.

**Table 6:** The top 30 hot keywords.

No.	Keyword	Frequency	Centrality	No.	Keyword	Frequency	Centrality
1	Psychoanalysis	36	0.59	16	Patient	3	0.05
2	Winnicott	17	0.16	17	Transitional objects	3	0.08
3	Transitional space	7	0.06	18	Field	3	0.03
4	Basic concepts	5	0.01	19	Object relations	3	0.15
5	potential space	5	0.04	20	Borderline personality disorder	3	0.08
6	Experience	4	0.01	21	History of psychoanalysis	3	0.03
7	Analytic theory	4	0.07	22	False self	3	0.02
8	Attachment	4	0.12	23	Depression	3	0.02
9	Anna Freud	4	0.03	24	Social work	3	0.01
10	Transitional object	4	0.03	25	Transitional phenomena	3	0.04
11	Emotions	4	0.02	26	Enactment	3	0.01
12	Counter transference	4	0.01	27	Holding environment	3	0.06
13	Self	3	0.01	28	Psychotherapy	3	0.12
14	Art	3	0.01	29	Death drive	3	0.01
15	Therapeutic action	3	0.04	30	Object	3	0

### 7.6. Hot topics of keyword clusters

According to the keywords clustering information, the 14 key keywords clusters can be summarized into two categories of hot topics.

The first category of research focuses on Winnicott’s related views of psychoanalytic therapy and object relation theory and the application of Winnicott’s theory, including cluster #0 potential space, cluster #1 donald winnicott, cluster #4 psychotherapy, cluster #6 transitional object, cluster #8 holding environment, and cluster #21 role playing.

In this category, many scholars have studied the application of Winnicott’s theory in psychoanalytical therapy or related fields. For instance, J. F. Rabin (15) contends that, due to his personal history of childhood, the dual experience of child observation and analysis enabled Winnicott to introduce the maternal environment into the treatment and to develop a theoretical framework, which describes the psyche of the baby has a close relationship with the maternal psyche; Steven Groarke (16) elaborates the clinical significance of Winnicott’s interpretation of trauma and its aftermath. He links the management of regression in the analytic setting to Winnicott’s theory of being, and he puts forward the redemptive potential between psychoanalysis and poetry by particular reference to the relationship between Winnicott’s work and that of T. S. Eliot; John Thor Cornelius (17) connects neuroscience with psychoanalysis by integrating Winnicott’s theories of psychic spaces with the neuroscientific examinations of patients with bilateral hippocampal injury; Susan Linn (18) has used puppet shows to help children facing serious illness and hospitalization based on Winnicott’s playing therapy theory; Martin Lawes (19) proposes an approach of improvisation-based music therapy, referring to Winnicott’s theory of gaming, creativity, and psychological therapy. Luca Quagelli (20) makes a deep exploration of Winnicott’s famous notion of regression to dependence. He elaborates on both the creative potential and limitations of the concept, and conducts some psychoanalytic work with neurotic and psychotic children.

Some scholars focus on Winnicott’s theory of object relations and its application. For example, Yaakov Roitman (21) considers that Winnicott’s concept of survival and the use of the object can help the therapist find his identity and overcome the dissociative state so as to become an alive subject helping the patient; M. E. Oliva (22) applies Winnicott’s theory of object relations to language therapy, noting that language conversion can be used as a defensive function for bilingual clients and as a therapeutic tool for bilingual therapists. And among these types of research, views on Winnicott’s theory of transitional phenomena or objects are especially obvious. Some representative examples are worthwhile to be listed as follows. Ian Shaw (23), referring to Winnicott’s concept of “transitional space,” argues that video games are instrumental transitional space to understanding the everyday geographies of violence and warfare; Michael Ira Casher (24) believes that some of Winnicott’s ideas, such as holding environment and transitional object, have a profound influence on psychiatric theory and practice. His creative thinking is particularly relevant to the inpatient psychiatric setting. Tamsin Cottis (25) relates Winnicott’s paper “Transitional objects and transitional phenomena” (1953) to the work of integrative arts child psychotherapy, and he links the treatment effect with Winnicott’s ideas about creativity, aggression, motivation, and the expression of self. He points out, through the therapeutic use of objects, the children’s experiences of trauma can be addressed. Jeff Gordon (26) illustrates drama therapy to cure addiction based on Winnicott’s concept of transitional phenomenon.

The second category of research mainly focuses on the connection and difference between Winnicott’s psychoanalytic treatment theory and the classical psychoanalysis theory represented by Sigmund Freud, Anna Freud, and Melanie Klein including cluster #2 deliberative theology, cluster #3 neuroscience, cluster #5 British psychoanalytical society, cluster #7 transgenerational transmission, cluster #9 the difficult patient, cluster #10 psychodynamic psychotherapy, cluster #11 metaphor and cluster #14 complex. And the keywords of these clusters mainly involve Freud, the history of psychoanalysis, Klein, and British psychoanalytical society (Table 4).

In this type of research, lots of scholars focus on the analysis and comparison of Freud and Winnicott’s psychoanalytical theory in various fields. For example, M. Altmeyer (27) attempts to elaborate on the contradictions of the psychoanalytic concept of narcissism based on Winnicott’s views of the intersubjective genesis of self combining Freud’s definition of narcissism; Walker Shields (28) makes a comparison study between interpretation of the dream material and the principles of the theory of neuronal group selection according to the theories about dream interpretation of Freud and Winnicott. He suggests further study of the interplay between the domain of psychoanalysis and neurobiology. Vincenzo Bonaminio (29) discusses Freud’s notion of how the analyst’s individuality influences treatment as well as Winnicott’s ideas of how the analyst relates to the patient; Leopoldo Fulgencio (30) proposes that there are two kinds of referents for the term metapsychology: speculative and factual. While Winnicott has supplied metapsychology with factual foundations, he has rejected speculative metapsychology theorization; Christine Anzieu-Premmereur (31) uses Freud’s classical psychoanalytic concept and Winnicott’s theory to conduct the parent–child relationship intervention research. Ofra Eshel (12) points out that Winnicott has a revolutionary change in clinical psychoanalytic work. She suggests that Winnicott’s clinical revision of psychoanalytic work, which emphasizes regression in the treatment, has great significance especially; M. Nathan Szajnberg (32) proposes three categories of Winnicott’s technique and interpretations, and enunciates the peculiarity of Winnicott’s theories between traditional analysts; Vincenzo Bonaminio (33) highly recognizes Winnicott’s revolutionary place in the development of psychoanalytic theory, together with his important ideas as transitional space, the false self, and the use of the object; Michael Crocker (34) tries to analyze Freud and Winnicott’s psychoanalytical theory from a scientific perspective. He believes that Winnicott’s work has always remained under the shadow of Freud’s theory; Abram Jan (35) addresses that Winnicott’s clinical theory is significantly different from the psychoanalytical theory of Freud and Klein, and Winnicott’s concept of trauma is closely related to the change of early psychological parent–child relationship.

To sum up, seeing from the above visualization and comprehensive analysis, in terms of the research field and research hotspots, research of Winnicott’s theories focus on the application of his psychoanalysis theory and the comparison between his psychoanalytic therapeutic views and the classical psychoanalysis represented by Freud, which embodies worldwide authoritative recognition and attention.

## 8. Conclusion

Winnicott’s psychoanalytic treatment theory, formed through a lot of clinical practice, is far from the classical psychoanalytic treatment view represented by Freud. He emphasizes the influence of the environment and the key role of maternal upbringing in the early psychological growth of the individual. He promotes the use of transference and countertransference and the therapist’s holding in psychoanalysis. He advocates accepting patients’ emotions, meeting patients’ emotional needs, and emphasizing the emotional renurturing of patients so that they can feel real and existing, and gain the ability to connect with the real world. He believes that healthy individuals are not isolated, but will gradually become associated with the environment, and eventually form a relationship between individuals and the environment which can be called “interdependence.” Healthy individuals can relate to their environment (the outside world or the real world). Health means maturity, and human maturity, not only means personal growth, but is essentially a process of social adaptation (i.e., socialization). When being healthy, an adult can identify with society without sacrificing too much of his own spontaneity; in other words, an adult can take care of his own personal needs without sacrificing his obligations to society. And the health of society depends on the health of its members.

In contrast to the classical psychoanalytic treatment, which insists on maximum neutrality, Winnicott’s psychoanalytic theory is full of warmth and deeply touches people’s hearts. He keeps a wonderful balance between the emotion that psychoanalysis focuses on and the reason that science holds. His theory mostly derives from his clinical experience, and it seems, to some researchers, too much emphasis on practice and a lack of theoretical system. But just maybe because he has had the quality pragmatism of doctors and the free willfulness of artists, his theory can be medically practical, rational, and artistically sensitive. He has created a unique paradigm of psychoanalytic theory. With a deep sense of responsibility for society, Winnicott describes his landmark concepts, such as good-enough mother, facilitating environment, transitional object, etc., in plain daily language, and widely spreads them through lectures, broadcasts, and other mass media. His theory has affected generations of psychoanalysts and children caregivers, and nowadays his remarkable influence still amazingly lasts.

## Author contributions

ZX conceptualized and elaborated the present study. YY conducted the use of software and made statistical analysis of studies. KP gave consultation to the use of medical terms. All authors contributed to the article and approved the submitted version.

## Funding

This research was supported by the project of Social Science Foundation of Hunan Province “A Study of Growth Trauma in Lawrence’s Novels from the Perspective of Winnicott’s Growth Theory” (No. 17WLH37).

## Conflict of interest

The authors declare that the research was conducted in the absence of any commercial or financial relationships that could be construed as a potential conflict of interest.

## Publisher’s note

All claims expressed in this article are solely those of the authors and do not necessarily represent those of their affiliated organizations, or those of the publisher, the editors and the reviewers. Any product that may be evaluated in this article, or claim that may be made by its manufacturer, is not guaranteed or endorsed by the publisher.

## Supplementary material

Supplementary TABLE 1 Annual publications trend chart.

## References

[1] Winnicott DW. Playing and reality. London: Penguin Books (1971).

[2] Winnicott DW. Home is where we start from: Essays by a psychoanalyst. London: Penguin Books (1986).

[3] Winnicott DW. Holding and interpretation: Fragment of an analysis. New York: Hogarth (1986).

[4] Winnicott DW. The maturational process and the facilitating environment. London: Hogarth Press and the Institute of Psycho-Analysis (1965).

[5] Winnicott DW. Deprivation and delinquency. London: Tavistock Publications (1984).

[6] Winnicott DW. Collected papers: Through Paediatrics to psycho-analyses. New York: Basic Books, Inc (1958).

[7] Winnicott DW. Human Nature. London: Free Association Books (1988).

[8] Winnicott DW. The family and individual development. London: Tavistock Publications (1965).

[9] Winnicott DW. The child, the family and the outside world. London: Penguin Books (1964).

[10] Rudnytsky PL. Transitional objects and potential spaces: Literary uses of D. W. Winnicott. New York: Columbia University Press (1993).

[11] Xi H. The turning of the theory of object relations: A study of Winnicott. Fuzhou: Fujian Education Press (2008).

[12] Eshel O. From extension to revolutionary change in clinical psychoanalysis: the radical influence of Bion and Winnicott. Psychoanal Q. (2017) 86:753–94. doi: 10.1002/psaq.12169, PMID: 29235679

[13] Lenormand M. “Psychoanalysis partage”: Winnicott, the piggle, and the set-up of child analysis. Int J Psychoanal. (2018) 99:1107–28. doi: 10.1080/00207578.2018.1489709, PMID: 33951792

[14] Liu S Sun YP Gao XL Sui Y. Knowledge domain and emerging trends in Alzheimer’s disease: a scientometric review based on CiteSpace analysis. Neural Regen Res. (2019) 14:1643–50. doi: 10.4103/1673-5374.255995, PMID: 31089065 PMC6557102

[15] Rabin JF. Mother and child in the treatment. Rev Fran Psychanal. (1994) 58:839–54.

[16] Groarke S. Unthinkable experience: Winnicott's ontology of disaster and Hope. Am Imago. (2010) 67:399–429. doi: 10.1353/aim.2010.0020

[17] Cornelius JT. The hippocampus facilitates integration within a symbolic field. Int J Psychoanal. (2017) 98:1333–57. doi: 10.1111/1745-8315.12617, PMID: 28083959 PMC5655787

[18] Linn S. It’s not me! It’s him! Interactive puppet play to help children cope. J Appl Arts Health. (2020) 11:103–8. doi: 10.1386/jaah_00021_7

[19] Lawes M. On improvisation as dreaming and the therapist’s authentic use of self in music therapy. Br J Music Therapy. (2020) 34:6–18. doi: 10.1177/1359457519884047

[20] Quagelli L. Reading Winnicott: return to the concept of regression to dependence. Int J Psychoanal. (2020) 101:456–78. doi: 10.1080/00207578.2020.1754836 33945701

[21] Roitman Y. Intergenerational transmission of violence: shattered subjectivity and relational freedom. Psychoanal Soc Work. (2017) 24:144–62. doi: 10.1080/15228878.2017.1369439

[22] Oliva ME. A healing journey of the bilingual self: in search of the language of the heart. J Ethn Cult Divers Soc Work. (2019) 28:334–47. doi: 10.1080/15313204.2017.1384946

[23] Shaw I Graham R. Playing war. Soc Cult Geogr. (2010) 11:789–803. doi: 10.1080/14649365.2010.521855

[24] Casher MI. “There's no such thing as a patient”: reflections on the significance of the work of D. W. Winnicott for modern inpatient psychiatric treatment. Harv Rev Psychiatry. (2013) 21:181–7. doi: 10.1097/HRP.0b013e31828ea604, PMID: 24651506

[25] Cottis T. “You can take it with you”: transitions and transitional objects in psychotherapy with children who have learning disabilities. Br J Psychother. (2017) 33:17–30. doi: 10.1111/bjp.12268

[26] Gordon J. Clown therapy: a drama therapy approach to addiction and beyond. Arts Psychother. (2018) 57:88–94. doi: 10.1016/j.aip.2017.12.001

[27] Altmeyer M. Narcissism, intersubjectivity, recognition. Psyche. (2000) 54:143–71.

[28] Shields W. Dream interpretation, affect, and the theory of neuronal group selection: Freud, Winnicott, Bion, and Modell. Int J Psychoanal. (2006) 87:1509–27. doi: 10.1516/31TQ-500Q-UKDL-YKEA, PMID: 17130080

[29] Bonaminio V. The person of the analyst: interpreting, not interpreting and countertransference. Psychoanal Q. (2008) 77:1105–46. doi: 10.1002/j.2167-4086.2008.tb00377.x, PMID: 18942500

[30] Fulgencio L. Discussion of the place of metapsychology in Winnicott's work. Int J Psychoanal. (2015) 96:1235–59. doi: 10.1111/1745-8315.12313, PMID: 25885643

[31] Anzieu-Premmereur C. Using psychoanalytic concepts to inform interpretations and direct interventions with a baby in working with infants and parents. Int Forum Psychoanal. (2016) 26:54–8. doi: 10.1080/0803706X.2016.1195512

[32] Szajnberg N. The piggle: decoding an enigma. Br J Psychother. (2017) 33:470–91. doi: 10.1111/bjp.12327

[33] Bonaminio V. Clinical Winnicott: traveling a revolutionary road. Psychoanal Q. (2017) 86:609–26. doi: 10.1002/psaq.12159, PMID: 28815713

[34] Crocker M Baur A. Connecting loose ends: integrating science into psychoanalytic theory. Clin Soc Work J. (2020) 50:1–11. doi: 10.1007/s10615-020-00774-9

[35] Jan A. On Winnicott's concept of trauma. Int J Psychoanal. (2021) 102:778–93. doi: 10.1080/00207578.2021.1932079 34196260

